# Partial dechlorination of 2,4,4′-trichlorobiphenyl (PCB 28) mediated by recombinant human CYP1A2

**DOI:** 10.1007/s00204-023-03621-1

**Published:** 2023-11-02

**Authors:** Isabella Randerath, Natalia Quinete, Julian Peter Müller, Julia Stingl, Jens Bertram, Thomas Schettgen, Thomas Kraus, Patrick Ziegler

**Affiliations:** 1 Institute for Occupational, Social and Environmental Medicine, Medical Faculty, RWTH Aachen University, Pauwelsstrasse 30, 52074 Aachen, Germany; 2 Department of Chemistry and Biochemistry, Institute of Environment, Florida International University, 3000 NE 151st Street, North Miami, Florida, 33181 USA; 3 Institute of Clinical Pharmacology, University Hospital of RWTH, 52074 Aachen, Germany

**Keywords:** Polychlorinated biphenyls, PCB28, Cytochrom p450 Monooxidase, Dechlorination

## Introduction

Polychlorinated Biphenyl (PCB) congeners show a long persistence in the human body with elimination half-lives in the range of 1–20 years. Elimination involves xenobiotic-metabolizing Phase I (cytochrome P450, i.e., CYP) and conjugating Phase II enzymes, which can lead to the formation and retention of OH-PCBs, PCB sulfates, PCB glucuronides, and PCB methyl sulfones (MeSO_2_-PCBs) (Grimm et al. 2015). Metabolic profiles of PCB degradation are dramatically affected by chlorine substitution patterns with implications for PCB toxicity and elimination half-live. Dechlorination of PCBs is considered to detoxify higher-chlorinated PCBs and has been demonstrated in animal models and in human cell culture (Tulp et al. 1977) (Zhang et al. 2022).

PCB28 is one of the quantitatively most important congeners of industrial PCB mixtures. Together with other lower-chlorinated PCBs (e.g., PCB52 and PCB101), it can be regularly detected in contaminated indoor environments (Schettgen et al. 2012; Kraft et al. 2018) and is found in the recycling of transformers and capacitors (Schettgen et al. 2011). There is experimental evidence from PCB28 that indicates a direct genotoxic effect and thus hints to possibly a direct carcinogenic effect (Vasko et al. 2018). We here show for the first time the partial dechlorination of PCB 28 to the dichlorinated monohydroxylated metabolite 3-OH-CB15, which is formed by human CYP1A2 from PCB28 in addition to its already known trichlorinated metabolites.

## Material and methods

### Analytical standards used in this study

The analytical standards of the trichlorinated PCB28 metabolites 2,4,4′-trichloro-5-biphenylol (5-OH-PCB28), 2′,3′,4-trichloro-4′-biphenylol (4′-OH-PCB25), and 2,4′,5-trichloro-4-biphenylol (4-OH-PCB31) were custom synthesized at the Max Planck Institute for Biophysical Chemistry, Facility for Synthetic Chemistry (Göttingen, Germany). The characterization was performed via NMR spectroscopy and mass spectrometry (data not shown). 2,4,4′-trichloro-3′-biphenylol (3′-OH-PCB28) was purchased from Combi-Blocks (San Diego, CA, USA). 4,4′-dichloro-3-biphenylol (3-OH-PCB15) was purchased by Chem Service (West Chester, PA, USA). For quantifications an internal standard of ^13^C_6_—3′-OH-PCB28 was custom synthesized and characterized via NMR spectroscopy and mass spectrometry (Göttingen, Germany).

### Incubation of PCB28 with recombinant CYP1A2 and CYP1A2 expressing transgenic HEK293 cells

Recombinantly expressed CYP1A2 bactosomes (coexpressed with CYP reductase in Escherichia coli) were obtained from Tebu-Bio (Offenbach, Germany) and mixed in the concentrations described. Incubations contained PCB 28 (20 µM), HEPES buffer (50 mM pH 7.4), MgCl_2_ (30 mM), NADPH (1 mM), and 5 pmol of bactosomes in a total volume of 500 µL. Incubations were performed in triplicates and initialized by adding NADPH after 3 min of pre-incubation at 37 °C and terminated after 60 min. Control samples were run in the absence of substrate, in the absence of NADPH or using heat-deactivated enzymes (Idda et al. 2020). For the incubation of PCB28 with CYP1A2 expressing transgenic HEK293 cells, 4 × 10^5^ cells were cultured in Dulbecco’s Modified Eagle Medium (DMEM) supplemented with 10% (vol/vol) fetal calf serum (FCS), penicillin (50 U/mL), and streptomycin (50 μg/ml) in a total volume of 2.5 ml. At a confluence of 80%, PCB 28 (20 µM) was added and cells were incubated for 24 h before harvest. Control samples were run with non-transgenic HEK293 cells.

### Analysis of PCB metabolites

Supernatants from bactosome incubations or cell culture experiments were diluted 1:2 with 80 µL acetate buffer 0.1 M (pH = 5.3). 100 µL of this dilution was further incubated with 100 µL of ammonium acetate buffer 0.1 M (pH = 5.3) and 5 µL of ß-Glucuronidase/Arylsulfatase enzyme overnight in a drying oven at 37 °C for enzymatic hydrolysis in order to release conjugated compounds. 50 µL of a mix of internal standards (10 ng mL^−1^) and 600 µL of methanol were added to the samples, then mixed by vortexing for 1 min, and centrifuged for 10 min at 4500 rpm. The individual supernatants were transferred to glass LC vials and evaporated to approximately 50 µL at 45 °C under a gentle stream of nitrogen. Finally, 0.1 mol L^−1^ ammonium acetate buffer was added to a final volume of 100 µL and then transferred to an insert for analysis. The online solid phase extraction (SPE) method coupled to liquid chromatography-tandem mass spectrometry was carried out using an API 5500 QTrap mass spectrometer (AB Sciex, Darmstadt, Germany) equipped with electrospray ionization (ESI) interface (Quinete et al. 2015).

## Results and discussion

**Fig. 1:**
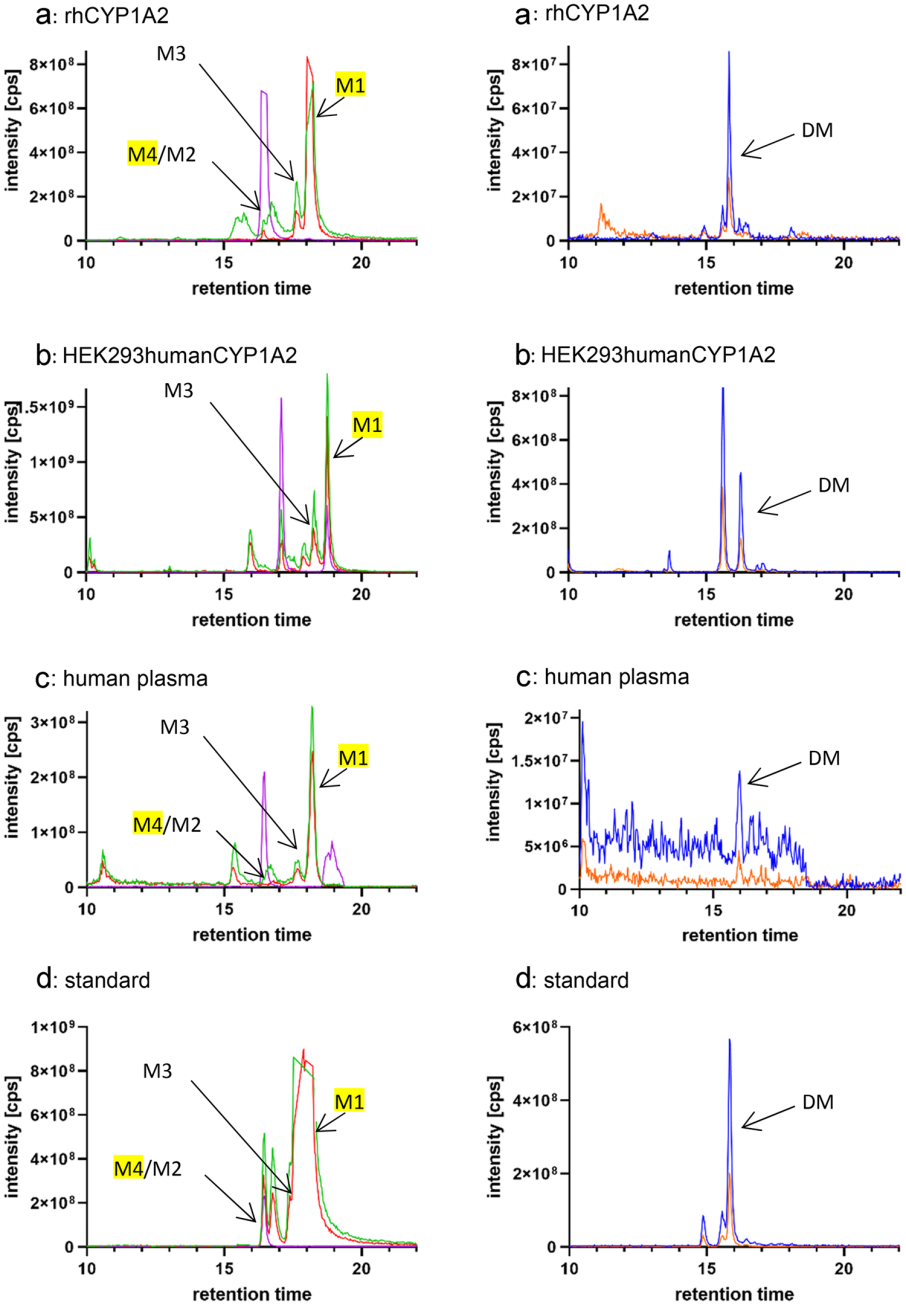
The extracted ion chromatograms of trichlorinated (left panel) and dichlorinated (right panel) OH metabolites of PCB28. **a** RhCYP1A2 expressed in E.coli (bactosomes) was incubated with PCB 28 for 1 h. **b** HEK293humanCYP1A2 cell line was incubated with 20 µM PCB28 for 24 h with subsequent harvest of the supernatant. **c** human plasma sample of a PCB28 exposed individual. **d** Standards. Representative images from 3 different experiments are shown. M (metabolite 1–4), DM (Dichlorinated Metabolite). Mass transitions are related to the following metabolites: trichlorinated 3′-OH-PCB28, 5-OH-PCB28, 4′-OH-PCB25, 4-OH-PCB31 m/z: 271.1- > 235.0 green, m/z: 273.2—> 237.0 red, m/z: 277.1—> 241.0 pink (the last one equalizing the internal standard ^13^C_6_-3′-OH-PCB28); dichlorinated 3-OH-PCB15):m/z: 237.2—> 201.0 blue, m/z: 239.2—> 202.9 orange (color figure online)

**Fig. 2:**
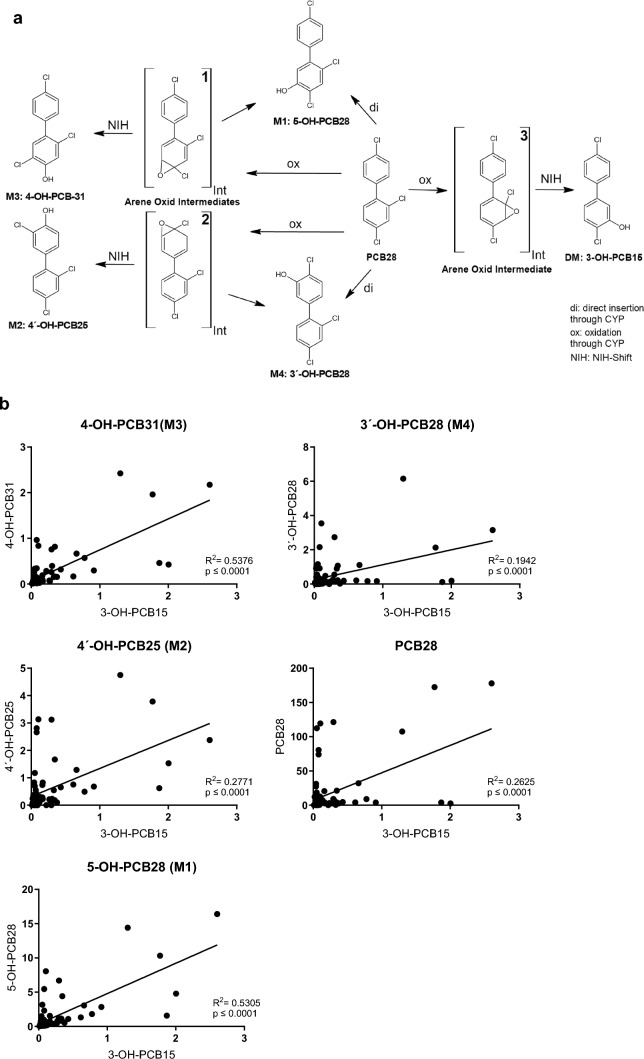
**a** Proposed scheme of the PCB28 pathway: transition states 1 and 2 lead to known trichlorinated OH metabolites M1– M4 (Quinete et al.), transition state 3 could lead to dechlorination of PCB28 and formation of 3-OH-PCB15. **b** Linear regression of plasma concentrations (*n* = 74; µg/L) of PCB28, 5-OH-PCB28, 4-OH-PCB31, 4′-OH-PCB25, and 3′-OHP-PCB28 versus plasma concentrations of 3-OH-PCB15

The metabolism of PCB28 was analyzed in vitro using recombinant human (rh)CYP1A2 expressed in E.coli (bactosomes), producing several monohydroxylated trichlorinated metabolites identified as 5-OH-PCB28 (M1, RT 18.76 min), 4-OH-PCB31 (M3, RT 18.28 min), and 3′-OH-PCB28/ 4′-OH-PCB25 (M4/M2, RT 17.09 min) by comparison with the authentic standards (Fig. 1a and Table 1) and thereby corroborating with the results found in our previous studies. In addition, a prominent ion peak appeared in the chromatogram at a retention time of 16.25 min. A comparison with the standard compounds with the closest retention time suggested that this peak resembled the hydroxylated metabolite of 4,4′-dichlorobiphenyl (3-OH-CB15). To confirm metabolic origin of the above species, control experiments with no NADPH, no PCB28, or with heat inactivated bactosomes were conducted. Indeed, in the control experiments none of the metabolites were detected (Supplemental Fig. 1). These data suggested that CYP1A2 formed a partially dechlorinated hydroxylated metabolite of PCB28 presumably via the classical lower-chlorinated (LC) PCB metabolism pathway including arene oxide intermediate formation (Fig. 2a).

**Table 1:** Metabolites, experiment, and concentrations for each OH-PCB congener

Metabolite	Experiment	Concentration (µg/L)
3′-OH-PCB28/ 4′-OH-PCB25 (M4/M2)	HEK293human	0
	HEK293humanCYP1A2	0.21
	rhCYP1A2	0.11
	Human plasma	3.15/2.17
4-OH-PCB31 (M3)	HEK293human	0
	HEK293humanCYP1A2	0.07
	rhCYP1A2	0.12
	Human plasma	2.38
5-OH-PCB28 (M1)	HEK293human	0
	HEK293humanCYP1A2	1.02
	rhCYP1A2	2.14
	Human plasma	16.41
3-OH-PCB15 (DM)	HEK293human	0
	HEK293humanCYP1A2	0.16
	rhCYP1A2	0.10
	Human plasma	2.60

Former studies had shown that bactosomes might catalyze different reactions with different metabolites generated than with the corresponding enzymes expressed in eukaryotic cell cultures (Stiborová et al. 2017). To rule out the possibility that CYP1A2 mediated dechlorination of PCB28 is specific for the prokaryotic expression system, we generated a transgenic HEK293 cell line stably overexpressing the human cDNA of *CYP1A2*. The presence of genomically integrated *CYP1A2* cDNA cell clones was confirmed by polymerase chain reaction analysis (data not shown). Functional expression of CYP1A2 could be demonstrated by conversion of the common substrate caffeine to the specific paraxanthine product (Supplemental Fig. 2). Incubation of HEK293humanCYP1A2 cells with PCB28 leads to the formation of all 4 known monohydroxylated metabolites of PCB28 as expected, whereas in non-transgenic parental HEK293 cells metabolite formation could not be observed. In addition, HEK293humanCYP1A2 cells formed 3-OH-CB15 from PCB28 (Fig. 1b and Table 1).

To find out whether metabolic formation of 3-OH-PCB15 occurs also in vivo, we examined plasma samples from individuals in a cohort exposed to different PCB Aroclor mixtures (HELPcB cohort) (Kraus et al. 2012) for the presence of 3-OH-CB15. Indeed, we found this metabolite in samples selected for high PCB28 concentrations (Fig. 1c and Table 1). Moreover, the concentration of 3-OH-CB15 correlated mainly with the concentration of the major PCB28 metabolites 4-OH-CB31 and 3´-OH-CB28 (Fig. 2), both resulting from oxidation at the dichlorinated ring of the trichlorobiphenyl structure. In fact, there is indirect evidence (Song et al. 2013) that 3-OH-PCB15 is also produced as a metabolite of PCB15, a potential minor component of commercial PCB mixtures such as various types of Aroclor (Supplemental Table 1). Thus, as composition of Aroclor(s) to which the plasma donors were exposed to was not reported, we cannot completely exclude the possibility that 3-OH-PCB15 in their blood resulted from hydroxylation of PCB15.

We emphasize that––contrary to the general view that dechlorination of PCBs is accompanied by their detoxification––a more differentiated view must be taken in the case of the WHO indicator congener PCB28. For PCB15 the formation of a hydroquinone (PCB15-HQ) via dihydroxylated intermediates was clearly demonstrated in *vitro* (Song et al. 2013), and PCB15 also showed a mutagenic effect both in vitro and in vivo. Furthermore, PCB15 had a tumorigenic effect in an initiation–promotion experiment after a single administration to rats, with a 100-fold higher incidence of preneoplastic foci/cm3 in the liver (Espandiari et al. 2003). Even though its full extent is currently unclear in humans, the metabolism of PCB28 to hydroxylated PCB15 and further downstream hydroquinone metabolites should also be taken into account when assessing the potential risk of PCB mixtures.

## Data Availability

All data supporting the findings of this study are available within the paper and its Supplementary Information.
